# Endothelin Receptor Antagonists in Kidney Disease

**DOI:** 10.3390/ijms24043427

**Published:** 2023-02-08

**Authors:** Irene Martínez-Díaz, Nerea Martos, Carmen Llorens-Cebrià, Francisco J. Álvarez, Patricia W. Bedard, Ander Vergara, Conxita Jacobs-Cachá, Maria José Soler

**Affiliations:** 1 Nephrology and Transplantation Research Group, Vall d’Hebron Institut de Recerca (VHIR), Vall d’Hebron Hospital Universitari, Vall d’Hebron Barcelona Hospital Campus, Passeig Vall d’Hebron 119-129, 08035 Barcelona, Spain; 2 Travere Therapeutics, Inc., San Diego, CA 92130, USA

**Keywords:** endothelin, endothelin receptor antagonists (ERAs), atrasentan, sparsentan, kidney disease

## Abstract

Endothelin (ET) is found to be increased in kidney disease secondary to hyperglycaemia, hypertension, acidosis, and the presence of insulin or proinflammatory cytokines. In this context, ET, via the endothelin receptor type A (ET_A_) activation, causes sustained vasoconstriction of the afferent arterioles that produces deleterious effects such as hyperfiltration, podocyte damage, proteinuria and, eventually, GFR decline. Therefore, endothelin receptor antagonists (ERAs) have been proposed as a therapeutic strategy to reduce proteinuria and slow the progression of kidney disease. Preclinical and clinical evidence has revealed that the administration of ERAs reduces kidney fibrosis, inflammation and proteinuria. Currently, the efficacy of many ERAs to treat kidney disease is being tested in randomized controlled trials; however, some of these, such as avosentan and atrasentan, were not commercialized due to the adverse events related to their use. Therefore, to take advantage of the protective properties of the ERAs, the use of ET_A_ receptor-specific antagonists and/or combining them with sodium-glucose cotransporter 2 inhibitors (SGLT2i) has been proposed to prevent oedemas, the main ERAs-related deleterious effect. The use of a dual angiotensin-II type 1/endothelin receptor blocker (sparsentan) is also being evaluated to treat kidney disease. Here, we reviewed the main ERAs developed and the preclinical and clinical evidence of their kidney-protective effects. Additionally, we provided an overview of new strategies that have been proposed to integrate ERAs in kidney disease treatment.

## 1. Introduction: The Endothelin System

Endothelin (ET) is a 21-aminoacid polypeptide described as the major vasoconstrictor of the organism. It is produced mainly by endothelial cells, but also by cells of the renal system, such as the epithelial and mesangial cells [1]. Hickey et al. were the first to describe the existence of a molecule capable of causing capillary constriction produced by the endothelium in 1985 but it was not until 1988 that ET was identified [2,3]. The ET polypeptide is present in three isoforms: ET-1, ET-2 and ET-3, with ET-1 being the greatest vasoconstrictor and the only one found at the protein level in the kidney [4]. ET-2 and ET-3 differ from ET-1 in two and five residues of the N-terminal end, respectively (Table 1), which determines the differences on the receptor-binding affinity [5]. Moreover, ET-1 is mainly released by endothelial cells, while the intestine and the kidney produce ET-2 and the neural tissue releases ET-3; the three isoforms can act in a paracrine or autocrine manner [5].

The action of ET is channelled through two membrane G-protein coupled receptors: Endothelin receptor A (ET_A_) and B (ET_B_). ET_A_ is localized in vascular smooth muscle cells and presents more binding affinity for ET-1 and ET-2 than for ET-3, due to the differences in the amino acid sequences (Table 1). ET_A_ activation induces a robust vasoconstrictor response and promotes cell proliferation and accumulation of the extracellular matrix. ET_B_ is present in vascular smooth muscle cells and endothelial cells. The three ET isoforms present the same affinity for the ET_B_ receptor, and its activation produces antiproliferative and antifibrotic effects, as well as the release of various vasodilator molecules [6]. Some experts have suggested an extended classification of the ET receptors, subdividing ET_B_ into ET_B1_ and ET_B2_ to differentiate the receptors present on the endothelial cells and the ones present on the smooth muscle cells, respectively. Nonetheless, there is no pharmacological evidence that demonstrates a difference between the receptors expressed by these two cell types [7]. ET-1 binding to ET_A_ causes G-proteins and phospholipase C (PLC) to join, leading to inositol triphosphate (IP_3_) and diacylglycerol (DAG) formation. Then, IP_3_ activates specific endoplasmic reticulum receptors to stimulate the release of stored Ca^2+^ causing a fast increase in intracellular Ca^2+^, which allows cell contraction and subsequent vasoconstriction. ET-1 activity through ET_A_ receptors also involves other signalling pathways, such as the phospholipase D (PLD) or mitogen-activated protein kinase (MAPK) pathway, to carry out other physiological effects such as cell growth or mitogenesis [5]. Contrarily, ET_B_ receptors produce their vasodilator effects through the activation of the nitric oxide synthases (NOS) system and the release of vasodilators as nitric oxide (NO) [8,9].

**Figure 1:**
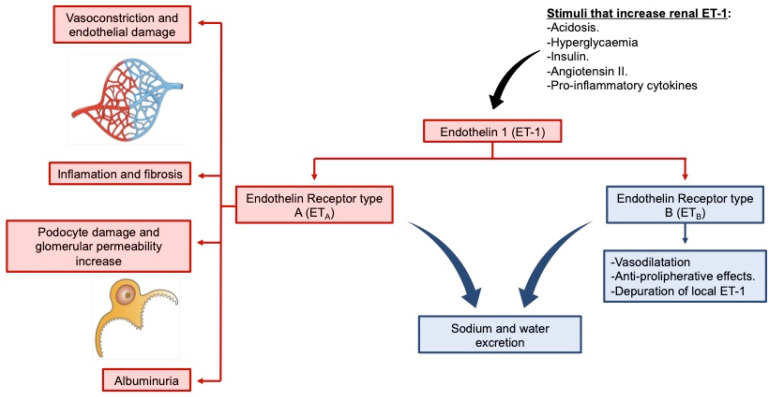
Scheme of the endothelin system. ET-1 acts thought its binding to ET_A_ and ET_B_ producing opposite effects in the kidney. The effects caused by the activation of ET_A_ are shown in red and the effects of ET_B_ activation are shown in blue. In pathological conditions, the hyperglycaemia, acidosis and the presence of insulin, angiotensin II and proinflammatory cytokines causes the increase of ET-1 concentration, which produces deleterious effects on renal function, such as vasoconstriction and endothelial damage, inflammation, fibrosis, podocyte damage or albuminuria.

In the kidney, ET has an essential role in blood flow and glomerular filtration regulation and in water–sodium and acid–base balances. ET_A_ and ET_B_ are expressed on the glomerular podocytes, mesangial cells and on the afferent and efferent arterioles. Regarding the tubular compartment, ET_B_ is expressed in all the regions in the renal tubule while ET_A_ is scarcely expressed on the proximal tubule and the descending Henle’s loop [10]. In physiological conditions, ET-1 through ET_A_ produces vasoconstriction of the afferent arteriole, reducing blood flow and, consequently, the glomerular filtration rate (GFR). Contrarily, the activation of ET_B_ induces vasodilation, antiproliferative effects and ET-1 depuration [8,9]. In pathological conditions, such as diabetes or hypertension, the concentration of ET-1 is increased because of the hyperglycaemia, acidosis and the presence of insulin, angiotensin II and proinflammatory cytokines, which causes sustained vasoconstriction. This may contribute to deleterious effects such as hyperfiltration (mainly in early diabetic nephropathy or incipient obesity-related kidney disease [11,12,13]) or podocyte damage and, eventually, proteinuria and GFR decline (Figure 1) [14].

The endothelin receptor antagonists (ERA) are postulated as a therapeutic strategy to reduce proteinuria and delay the progression of GFR decline [14]. Promising results using ERAs in kidney disease have been obtained in recent years. The purpose of this review is to provide an overview of the main ERAs and of the mechanisms by which these drugs protect the kidney with a special focus on the results obtained in updated experimental studies and randomized clinical trials. Additionally, we provide a glance of the main novel approaches to introduce ERAs for kidney disease prevention.

## 2. Methods

We searched PubMed, Scopus and Google academic during November-December 2022 using the following search terms (alone of combined) to find publications related to the endothelin system and the endothelin receptor antagonists in experimental and human kidney disease: “endothelin or ET”, “endothelin receptor antagonists or ERA”, “endothelin receptor A or ET_A_”, “endothelin receptor B or ET_B_”, “kidney”, “kidney disease”, “chronic kidney disease”, ”kidney injury”, “experimental models”, “mice”, ”rat”, “podocytes”, “randomized clinical trials”. We critically reviewed the reports found and selected the relevant studies to construct the review text. For the sections “5. Preclinical experimental evidence of ERAs protective effects on kidney damage” and “6. Randomized controlled trials (RCTs) using ERAs for prevention of kidney disease progression”, we mainly focused on the works published in the last five years to not overlap previous reviews on the topic. The complete literature review strategy is available from the authors upon request. Further, we consulted https://clinicaltrials.gov during November–December 2022 to obtain information regarding unpublished ongoing clinical trials that we have stated using the corresponding National Clinical Trial (NCT) number.

## 3. The Endothelin Receptor Antagonists

Endothelin receptor antagonists (ERAs) are drugs that block the endothelin receptors, preventing the endothelin action. There exist different types of ERAs, which can be distinguished by their affinity for binding to ET_A_ or ET_B_. In some cases, these antagonists do not present selectivity and are able to interact with both receptors. The selectivity of an ERA for each receptor subtype is determined by a competition binding assay against [125I]-ET-1 that allows to calculate the equilibrium dissociation constant of each compound to both receptors, ET_A,_ and ET_B_. To establish a selectivity threshold, in 2006, Maguire and Davenport et al. [15] proposed that an ERA should present more than 100-fold selectivity for ET_A_ or ET_B_ to be considered selective for one or the other receptor. Those with less than 100-fold selectivity should be classified as non-selective or mixed antagonists [15]. The main ERAs are summarized in Table 2. 

The majority of the antagonists here described are still under investigation in ongoing clinical trials, while others are not used in clinical practice because of lack of efficacy or due to the presence of adverse events related to their use that compromise the safety of patients. Therefore, to take advantage of these compounds with clear beneficial effects (Table 2), therapeutic approaches under study are the combination of ERAs with other nephroprotective drugs such as sodium-glucose cotransporter 2 inhibitors (SGLT2i) and the use of dual drugs such as sparsentan that blocks at the same time angiotensin-II type 1 and endothelin receptors. 

### 3.1. ET_A_-Selective Receptor Antagonists

The binding of ET-1 to ET_A_, in pathologic conditions, can lead to vasoconstriction, inflammation, cellular injury, fibrosis, and, finally to proteinuria and loss of renal function [31]. To counteract these effects, several selective ET_A_-receptor antagonists have been developed as potential therapeutic agents. Some of them are currently used to treat pulmonary arterial hypertension (ambrisentanand macitentan), meanwhile others are still under study in ongoing randomized clinical studies. To date, none of the ET_A_-selective receptor antagonists have been approved to treat kidney disease, despite their demonstrated kidney protective effects (Table 2). 

BQ-123 was the first ET_A_ selective antagonist peptide isolated that derived from *Streptomyces misakiensis* fermentation products. It has been used in investigation in both animals and humans where the molecule reduced glomerular permeability [20]. Darusentan (LU 135252) is a selective endothelin receptor antagonist, with high affinity towards ET_A_ receptor [17], with a ratio of relative selectivity of 170:1 ET_A_:ET_B_ [32]. It is derived from the optimization of two initial lead structures (LU 110896 and LU 110897) found in a screening of the library of human recombinant ET_A_ receptors [33]. Darusentan was promising because reduced blood pressure in resistant hypertension patients in early clinical studies but unfortunately failed to achieve efficacy in phase III clinical studies [32]. Other examples of ETA selective antagonists are sitaxentan, ambrisentan, avosentan, atrasentan, macitentan and zibotentan (Table 2). Currently, sitaxentan, ambrisentan and macitentan are approved to treat pulmonary arterial hypertension [21,34,35] while avosentan, atrasentan and zibotentan have been proposed as therapeutic agents in kidney disease [19,24,36]. Macitentan is a sulfamide with high affinity for ET_A_ that has been used in pulmonary arterial hypertension since its approval in 2013 [37,38]. It belongs to the next generation of antagonists, as it was developed following the structural basis of bosentan [20], but with improvements such as a prolonged receptor binding capacity and better pharmacodynamics and pharmacokinetics [37,38]. In vivo, macitentan is metabolized by cytochrome P450 3A4 (CYP3A4) into an active metabolite, which is called aprocitentan (a non-selective ERA, see below) [26]. Avosentan is a selective ET_A_ inhibitor which presents ~500-fold selectivity for ET_A_ over ET_B_ receptor [39]. It was developed for the treatment of diabetic nephropathy. A study performed by Wenzel et al. in 2009 [23] demonstrated that the addition of avosentan to the standard of care antihypertensive therapy with RAS blockers produced additional antiproteinuric effects in diabetic nephropathy patients. However, a second study to test long-term treatment with avosentan was stopped prematurely because of safety concerns [36]. Atrasentan is an oral selective ET_A_ inhibitor with a selective ET_A_:ET_B_ blockade ratio of 1200:1 [40] and 1800-fold selectivity for ET_A_ [39]. In patients with diabetes and chronic kidney disease, atrasentan reduces the risk of renal events and albuminuria [19,22,41]. Currently, it is under study in an ongoing phase 2 clinical trial to evaluate the efficacy and safety of atrasentan in patients with proteinuric glomerular diseases (AFFINITY: Atrasentan in Patients with Proteinuric Glomerular Diseases—NCT04573920). Finally, zibotentan is a selective ET_A_ antagonist, which shows a potent affinity to this specific receptor [42]. In a recent clinical trial, it has been demonstrated that zibotentan could be beneficial for the treatment of systemic sclerosis-associated chronic kidney disease because its effects in the improvement of estimated GFR and the absence of increased endothelin serum levels during treatment [24].

### 3.2. ET_B_-Selective Receptor Antagonists

Few ET_B_-selective antagonists have been developed. This can be explained by the fact that when endothelin binds ET_B_, it triggers beneficial effects such as vasodilation; hence inhibiting the action of ET_B_ may not be a suitable therapeutic strategy. In addition, ET_B_-selective antagonists are usually less potent than ET_A_-selective agonists [20]. However, some small molecules have been developed to block ET_B_, such as non-peptide RO468443 that displays 2000-fold ET_B_ selectivity [43], and A192621 [20]. Nevertheless, the most important ET_B_-selective antagonist is BQ-788, which was described for the first time by Ishikawa et al. in 1994. BQ-788 has been studied in combination with BQ-123, an ET_A_-selective ERA, and it causes the reduction of glomerular permeability to albumin but does not add to the effect of BQ-123 in monotherapy [39]. In cancer, BQ-788 inhibits cell growth and induces the death of melanoma cells, both in vivo and in vitro [26].

### 3.3. Non-Selective Endothelin Receptor Antagonists

Some ERAs can interact with either ET_A_ or ET_B_ receptor. Some of the non-selective ERAs are bosentan, tezosentan and aprocitentan. Bosentan is a non-peptide derivative dual endothelin receptor antagonist with affinity to both receptors ET_A_ and ET_B_, but with barely higher affinity towards ET_A_ (ET_A_:ET_B_ 20:1) [22,35,40]. Currently it is used in the treatment of pulmonary arterial hypertension [34] and in paediatric idiopathic pulmonary hypertension [22]. Bosentan decreases vascular resistance, resulting in an increasing cardiac output without disrupting the heart rate. It also plays a role in the inhibition of endothelial cell proliferation [25]. Tezosentan is a dual endothelin receptor antagonist with a selectivity ratio of 30:1 ET_A_:ET_B_ [44]. It was developed for the treatment of heart failure and preclinical studies have shown that tezosentan improves hemodynamics and renal function in rats [26] but does not improve dyspnoea or reduce the risk of cardiovascular events [26]. Aprocitentan (ACT-132577) is a dual inhibitor of ET_A_/ET_B_ with a selective ratio of 1:16 [45,46,47]. It belongs to the sulfonamide class of molecules and is obtained by oxidative depropylation from macitentan [45,46,47]. In the PRECISION clinical trial, finished in 2022, aprocitentan lowered blood pressure in patients with resistant hypertension [27].

### 3.4. Other Types of Endothelin Receptor Antagonists

Sparsentan (BMS-346567) is a dual endothelin receptor/angiotensin-II type 1 receptor antagonist (DEARA) which presents high affinity for ET_A_ (~1000-fold). It was created by combining structural elements of both irbesartan, an angiotensin II type 1 receptor antagonist, and biphenylsulfonamide, an endothelin receptor antagonist. Thus, sparsentan blocks at the same time the RAS and the endothelin system reason why it is expected to show additive renoprotective effects. Sparsentan reduces blood pressure in hypertensive patients [28]. The antiproteinuric and the possible nephroprotective effects of sparsentan, are currently studied in focal segmental glomerulosclerosis (DUPLEX study) [29] and IgA Nephropathy (PROTECT study; NCT03762850) patients in an ongoing phase 3 clinical trials.

## 4. Mechanisms of Renal Protection Mediated by ERAs

ET, through the activation of its receptors, may be detrimental for the kidney, as it is involved in the progression of chronic kidney disease and other conditions such as diabetes [48]. Therefore, blockade of the ET receptors with ERAs has renal protective effects. ERAs protect the kidney by several mechanisms. First, this drug class has clear effects on glomerular hemodynamics [49,50,51]. ET_A_ receptor antagonism improves blood pressure via vasodilatation and decreases proteinuria and the filtration fraction (ratio of glomerular filtration rate over renal plasma flow), providing renoprotective effects [14]. Moreover, ET_A_ receptor blockade may improve endothelium-dependent relaxation and vasomotion [52,53,54]. There is no difference in terms of blood pressure reduction when comparing selective ET_A_ receptor antagonists and mixed ET_A_/ET_B_ receptors antagonists, which suggests that ET_B_ receptor blockade does not change blood pressure. This also implies that combined ET_A_/ET_B_ receptors antagonists and selective ET_A_ receptor antagonists are similar in terms of their hypotensive effects and ET_B_ receptor antagonists are not involved in this outcome [55]. Second, ERAs also produce effects on different renal cell types that express ET-1 or its receptors [56,57,58]. Podocytes are targets of ET-1 since they express ET_A_ [59]. In this sense, several studies have been focused on the effects of ET_A_ receptor antagonists on these cells. After treatment with ERAs, many studies have found a reduction in the podocyte injury, which lead to the stabilization of the glomerular and podocyte structure [60,61]. Exogenous ET-1 administration induced podocyte injury in rats, which could be prevented by ET_A_ receptor blockade [61]. Also, in a hypertensive rat model, selective ET_A_ receptor blockade restored podocyte injury and function. Further, ERAs ameliorate the structure of the glomerular basement membrane and have beneficial effects on glomerulosclerosis and proteinuria [62]. Mesangial cells produce ET-1, although in a much smaller proportion than endothelial cells. ET-1 produced by mesangial cells can act in an autocrine way by binding to ET receptors. Via ET_A_ it results in the contraction of mesangial cells, cell proliferation and mesangial matrix accumulation [63,64]. These deleterious effects can be blocked using ERAs [65,66]. As mentioned before, ET_B_ is expressed in all along the renal tubule but ET_A_ expression in the proximal tubule and the descending Henle’s loop is low [56]. ET_B_ receptor is responsible for the clearance of ET-1 and could have important implications since it modulates the presence of this vasoconstrictor [51]. Some studies reported that treatment with an ET_B_-selective receptor antagonist diminished ET-1 clearance, remaining in the plasma, and increasing the response to ET-1 leading to hypertension in some patients [67]. In addition to these effects, ET-1 can induce inflammation and fibrosis [16], since overexpression of ET-1 resulted in interstitial fibrosis in transgenic mice expressing human ET-1 [68] that can be reversed only by ET_A_-selective receptor antagonists [69].

In summary, ET_A_-selective ERAs show a wide range of renoprotective effects especially by ameliorating blood pressure and modulating kidney hemodynamics although, as mentioned, ERAs have beneficial effects not mediated by its blood pressure-lowering capacity. ERAs can restore podocyte injury and its function; improve mesangial matrix accumulation, inflammation, and fibrosis, eventually reducing glomerular permeability and proteinuria.

## 5. Preclinical Experimental Evidence of ERAs Protective Effects on Kidney Damage

In recent years, several preclinical studies have investigated the effects of ERAs using different experimental models. The first studies on cultured mesangial cells showed that ET produced cellular contraction, hypertrophy, and extracellular matrix production [70,71]. These effects were reversed using ERAs [66], as happened in hypertensive rats [72]. Experiments using stroke-prone spontaneously hypertensive rats (SHRSP) demonstrated that the ET_A_ receptor blockade provided renal protection by normalizing the expression of growth factors, diminishing extracellular matrix proteins, and reducing metalloproteinase-2 (MMP-2) activity [66]. Spires et al. studied the effect of atrasentan (an ET_A_ receptor antagonist, Table 2) in streptozotocin-treated Dahl salt-sensitive (STZ-SS) and type 2 diabetic (T2DN) rats. Both rat models showed increased levels of ET-1 during the progression of the renal disease. Atrasentan diminished glomerular injury and renal fibrosis in both models but only reduced arterial pressure and proteinuria in STZ-SS. This could be explained by differences in the severity of the kidney injury of these models [73]. In any case, it illustrates that improvement of kidney damage is possible without changing arterial pressure and/or proteinuria. In this sense, Harvey et al. [74], demonstrated that ET_A_ blocking with atrasentan (but not ET_B_ blocking) improved integrity and viability of cultured podocytes submitted to hypoxia to mimic chronic renovascular disease [74]. In this line, Dolinina et al. tested BQ-788 (an ET_B_ receptor antagonist, Table 2) and JKC-301 (an ET_A_ receptor antagonist) in Sprague-Dawley rats where glomerular permeability was induced by administration of ET-1. The study demonstrated that the glomerular hyperfiltration improvement was dependent on ET_A_ receptors, since ET_A_ receptor blockade ameliorated glomerular hyperfiltration but not ET_B_ receptor blockade [75]. Similar results were obtained in a study where the effects of BQ-788 and atrasentan were compared in uninephrectomized Sprague-Dawley rats on high-sodium diet (HS/UNX) and in spontaneously hypertensive rats (SHR). Both hypertensive rat models showed altered nitric oxide levels, possibly related to ET_A_ receptor hyperactivity. Also, in the HS/UNX model ET_A_ receptor blockade reduced blood pressure and decreased renal excretion, while ET_B_ receptor blockade did not alter blood pressure or renal excretion. The SHR model showed a reduction in blood pressure after treatment with atrasentan. This comparison confirmed, as well, the dependence of blood pressure and renal hemodynamics on ET_A_ receptors, since ET_B_ receptor antagonist did not modify renal hemodynamics [76]. Indeed, ET_A_ selective ERAs have clear renoprotective effects. In this sense sitaxentan (an ET_A_ receptor antagonist, Table 2) improved kidney function and tubular atrophy in a rat model of chronic interstitial nephritis induced by adenine. Further, sitaxentan in combination with cinacalcet (an allosteric modulator of the calcium sensing receptor), increased renal angiotensin converting enzyme 2 (ACE2) expression, which is protective for the kidney, and normalized urinary calcium loss [77]. Similar results were obtained in the study of Caires et al. who tested bosentan and macitentan (Table 2) in normotensive and hypertensive rats with cyclosporin A (CsA)-induced kidney damage. CsA is nephrotoxic and has a vasoconstrictive effect, which was partially reversed only by bosentan. However, both, bosentan and macitentan were able to improve the hemodynamic changes induced by CsA in hypertensive rats by decreasing blood pressure. Furthermore, bosentan and macitentan reduced the generation of reactive oxygen species produced by CsA. Thus, the ERAs used in these experiments had similar effects although bosentan seemed to be better at reversing the hemodynamic changes [78]. Ambrisentan (an ET_A_ receptor antagonist, Table 2) and bosentan showed similar kidney protective effects in an ischemia-reperfusion rat model in terms of reduction of kidney apoptosis, tissue damage and inflammation probably mediated by an increase of nitric oxide levels. Another study using the normotensive Wistar Kyoto (WKY) rats compared the effects of macitentan and sitaxentan. The results of these experiments revealed that sitaxentan prevented sunitinib-induced hypertension in the same manner as macitentan indicating that the increase in blood pressure was mediated by ET_A_ receptors. Sitaxentan also improved albuminuria and diminished prostacyclin levels [79].

The effects of ERAs have also been studied on top of RAS blockers, the current standard of care for many chronic kidney diseases [80,81,82]. Atrasentan combined with losartan (an angiotensin-II type 1 receptor blocker) improved podocyte number and structure and decreased proteinuria in BTBR ob/ob mice [83] similarly to what happens in human [19]. Other studies combined two RAS blockers (trandolapril and losartan) with atrasentan in a rat model of chronic kidney disease, showing an additional beneficial effect of the combination of ERAs with RAS blockers. This combination of drugs increased the survival rate and reduced proteinuria and renal glomerular damage [84]. Also, Gagliardini et al., combined avosentan (an ET_A_ receptor antagonist, Table 2) with lisinopril (an angiotensin converting enzyme inhibitor) in uninephrectomized streptozotocin-induced diabetic rats. Combined therapy was able to improve proteinuria, protected from glomerular and tubulointerstitial damage, restored podocyte number, nephrin levels and glomerular permeability. The combination also improved the deleterious changes in the peritubular capillaries and renal interstitial blood perfusion, which could lead to amelioration of the tubular function [85]. Preclinical studies in mice and rats using sparsentan, a new dual AT_1_/ET_A_ receptor antagonist with affinity to ET_A_ and angiotensin II (type 1) receptors (Table 2), showed that this dual inhibition protects the glomeruli from podocyte loss and podocyte foot effacement. The effects also include maintenance of the glomerulus basement membrane, glomerular glycocalyx integrity and reduction of blood pressure [86,87]. In addition, studies using multiphoton microscopy imaging in Confetti mice with focal segmental glomerulosclerosis induced by transient receptor potential channel 6 (TRPC6) overexpression, showed greater preservation of the kidney function in mice treated with sparsentan in comparison with the mice that received no drug or losartan [88]. ERAs have also been studied in combination with sodium-glucose type 2 cotransporter (SGLT2) inhibitors (SGLT2i) in several diabetic mice models because of the potential for SGLT2i to reduce the volume overload induced by ERAs. Atrasentan combined with dapagliflozin (an SGLT2i) did not improve albuminuria, glomerular filtration rate, kidney inflammation or fibrosis but ameliorated of glomerulosclerosis and podocyte injury in a mouse model of type 2 diabetic kidney disease. This suggests that the dual therapy approach can have therapeutic potential [89]. A recent study of Vergara, A. et al. [90] tested the capacity of an SGLT2 inhibitor (empagliflozin) and/or an ERA (atrasentan) on top of RAS blockade with ramipril to protect the diabetic kidney in experimental diabetic nephropathy using db/db mice. This study revealed that triple therapy with empagliflozin, atrasentan and ramipril maintained the impact of each therapy alone and added to organ protection. Empagliflozin combined with ramipril or in triple therapy with atrasentan ameliorated hyperfiltration, but only the triple combination exerted greater protection against podocyte loss. The combined therapy not only protected against kidney injury but also provided cardiac protection in terms of decrease of cardiomyocyte hypertrophy. Additionally, the add-on triple therapy further enhanced the intrarenal ACE2/Angiotensin(1-7)/Mas protective arm of the RAS. These data suggest that triple therapy with empagliflozin, atrasentan and ramipril have a synergistic cardiorenal protective effects in experimental diabetic nephropathy. Thus, the combination with RAS blockers and/or SGLT2i may promote the use of the ERAs in the clinical practice as it has shown add-on effects in experimental models and has the potential to mitigate adverse events produced by ERAs in monotherapy. This therapeutic approach is currently being evaluated in randomized controlled trials.

## 6. Randomized Controlled Trials (RCTs) Using ERAs for Prevention of Kidney Disease Progression

The largest trials testing ERAs have been performed in type 2 diabetic patients (Table 3). In these studies, ERAs have shown to reduce albuminuria and slightly decrease blood pressure [19,23]. The effect of selective endothelin antagonist on albuminuria is consistent across different studies, obtaining a 30–40% reduction on urine albumin-to-creatinine ratio (UACR) in the groups that received the active treatment. However, blood pressure reduction is moderate and shows different results between RCTs. Overall, selective ERAs seem to reduce 3–5 mmHg both systolic and diastolic blood pressure (SBP and DBP, respectively). The effects on BP vary among the employed ERA, with the greatest reductions described for darusentan (9.9 mmHg reduction in SBP and 4.6 mmHg reduction in DBP) [91]. Nevertheless, the latter study included patients with resistant hypertension, which may have contributed to the larger differences in the active treatment arms [91]. In addition, the SONAR study showed that BP reduction is more evident when initiating the treatment and becomes milder after chronic treatment [19].

Regarding GFR preservation, selective ERAs have displayed protective effects or no effect among the different RCTs performed to date. The SONAR trial, which treated responder patients (patients that showed a decrease in UACR of at least 30% with no substantial fluid retention during the enrichment period) for a median follow-up of 2.2 years, showed that 0.75 mg of atrasentan on top of the RAS blockade was able to preserve 0.65 mL/min/1.73 m^2^ of GFR and to prevent the doubling of serum creatinine during the treatment period [19]. In the same line, in patients with systemic sclerosis, zibotentan was able to preserve 4.3 mL/min/1.73 m^2^ of GFR after 6.5 months of treatment [24]. The only ERA that showed a significant decrease in GFR that could be related to the type of patients included and the greater BP reduction was darusentan [91].

When analyzing major renal events, only ASCEND and SONAR trials were designed to find differences in a primary composite kidney outcome [19,36]. The ASCEND trial had to be prematurely stopped because an increased number of deaths due to cardiovascular causes in the group of patients receiving the active treatment [36]. As death was included within the main composite outcome, the study was unable to find significant differences between the groups receiving avosentan and the group receiving placebo. The increased number of CV deaths was also linked to an increased number of adverse events: fluid overload, heart failure and anemia. However, if we only consider end-stage renal disease (ESRD) or doubling of serum creatinine as events, the group of patients receiving avosentan showed a lower risk compared to those treated with placebo (HR 0.63, 95%CI: 0.42–0.95). To overcome the evident adverse events related to the inhibition of ET_A_ receptor-mediated sodium and water excretion, the SONAR trial only included responder patients that did not show adverse events during an initial enrichment period [19]. In the latter study, atrasentan was able to reduce the number of renal events when compared to placebo. Nevertheless, previously described adverse events such as fluid overload, heart failure or anemia were again more frequent in the group treated with atrasentan. In this line, the addition of the new renoprotective SGLT2i to the treatment with ERAs in type 2 diabetic patients could prevent the development of fluid retention or anemia, as the former drug class has diuretic effects and increases hemoglobin levels [94,95]. A recent post-hoc analysis of patients receiving atrasentan and SGLT2i in the SONAR trial revealed that weight increase (a surrogate marker of fluid overload) was reduced in patients receiving both atrasentan and SGLT2i [96]. New trials with prespecified kidney outcomes that evaluate the synergistic effects of the combination will shed light upon the future of ERAs in the treatment of chronic kidney disease. The currently ongoing ZENITH-CKD trial (NCT04724837), for example, will evaluate the efficacy of the combination of zibotentan and dapagliflozin in the treatment of CKD.

Additionally, the use of ERAs is being extended to kidney diseases with albuminuria such as primary FSGS or IgA nephropathy, where the existence of previous cardiovascular comorbidities is less frequent and the risk of adverse events also lower. Sparsentan, a dual angiotensin II type 1 and endothelin type A (ET_A_) receptor antagonist, was already tested in the DUET trial that included patients with primary focal segmental glomerulosclerosis (FSGS) [97]. After eight weeks of treatment, sparsentan obtained greater reductions in proteinuria, and was superior to irbesartan, achieving partial remission of the disease (28% vs. 9%). The promising effects of sparsentan will be further confirmed by the ongoing trials on primary FSGS (DUPLEX study, NCT03493685) and IgA nephropathy (PROTECT and SPARTAN studies, NCT03762850 and NCT04663204, respectively). The ALIGN (NCT04573478) will also give insights about the impact of the combination of atrasentan and RAS blockade for the treatment of IgA nephropathy.

## 7. Conclusions

As reviewed here, the concentration of ET-1 is increased in pathological conditions, such as diabetes or hypertension, causing sustained vasoconstriction that ultimately leads to kidney damage. The ERAs show clear renoprotective effects in preclinical experimental models and in human mainly by hemodynamic effects but also by restoring podocyte injury, reducing mesangial matrix accumulation, fibrosis and inflammation which reduces glomerular permeability and proteinuria. However, the use of ERAs in clinical practice to prevent kidney disease is narrow because some ERAs failed to demonstrate efficacy in phase III randomized clinical trials and/or produced adverse events such as oedemas. To overcome these limitations, the combination of ERAs with SGLT2i have been proposed as well as the use of the dual angiotensin-II type 1/endothelin receptor blockers. The utility of these therapeutic approaches to treat kidney disease is currently being tested in ongoing randomized controlled trials.

## Figures and Tables

**Table 1:** ET1, ET2 and ET3 amino acid sequences. ET is a 21-amino acid peptide present in three different isoforms in the organism: ET-1, ET-2 and ET-3 [5]; the differences in amino acid sequence between them are shown in bold.

ET Isoform	Amino Acid Sequence
ET-1	NH_2_—CSCSSLMDKECVYFCHLDIIW—CH_3_
ET-2	NH_2_—CSCSS**WL**DKECVYFCHLDIIW—CH_3_
ET-3	NH_2_—C**T**C**FTYL**DKECVYFCHLDIIW—CH_3_

**Table 2:** Main endothelin receptor antagonists (ERAs), the selectivity for the ET receptors, year of development, the current status and the protective effects of each of them.

ERA	Affinity	Year of Development ^¥^	Status ^¥^	Protective Effects	Diseases Tested	References
BQ-123	ET_A_	1995	--	Potent vasodilatation in the peripheral circulation Reduction of albumin permeability	--	Saleh, M., et al., 2010 [16]
Darusentan	ET_A_ ~1000-fold	1996	Investigational	Inhibition of vasoconstriction	Resistant Hypertension Chronic Heart Failure	Liang, F., et al., 2010 [17]
Atrasentan	ET_A_ ~1800-fold	1996	Investigational	Reduction of albuminuria Risk of renal events	Diabetic Nephropathy	de Zeeuw, D., et al., 2014 [18] Heerspink, H., et al., 2019 [19] Davenport A.P. et al., 2016 [20]
Sitaxentan	ET_A_ ~6500-fold	1997	--	Reduction of tubular atrophy Influences kidney hemodynamics and improves kidney function	Pulmonary Arterial Hypertension Chronic Renal Insufficiency	Scott, L.J., et al., 2007 [21]
Ambrisentan	ET_A_ >4000-fold	2004	Approved in 2007	Inhibition of vasoconstriction effects	Pulmonary Arterial Hypertension	Enevoldsen, F.C., et al., 2020 [22]
Macitentan	ET_A_ ~1000-fold	--	Approved in 2013	Stimulate vasodilation, Marked antitumoral effects in an experimental model of multidrug-resistant ovarian tumors	Pulmonary Arterial Hypertension	Davenport A.P., et al., 2016 [20]
Avosentan	ET_A_ ~500-fold	2006	Investigational	Reduction of albuminuria	Diabetic Nephropathy	Wenzel, R.R., et al., 2009 [23]
Zibotentan	ET_A_	2010	Investigational	Improvement in eGFR Not evidence of an increment of serum endothelin levels Reduction of blood pressure	Chronic Kidney Disease Potential use for Alzheimer Disease Heart failure, hormone resistant prostate cancer and other cancers	Stern, E.P., et al., 2022 [24]
BQ-788	ET_B_	1994	--	Reduction of albumin permeability in combination with BQ-123	--	Saleh, M., et al., 2010 [16]
Bosentan	ET_A_/ET_B_	1999	Approved in 2001	Decrease vascular resistance Inhibition of endothelial cells proliferation	Pulmonary Arterial Hypertension	Wang, Y., et al., 2019 [25]
Tezosentan	ET_A_/ET_B_	2001	--	Decrease serum creatinine Increases GFR Maintained renal architecture in kidneys after ischemia	Pulmonary Arterial Hypertension Acute Heart Failure	Mekuria, et al., 2021 [26]
Aprocitentan	ET_A_/ET_B_	2015	Investigational	Decrease blood pressure	Resistant Hypertension	Schlaich, M.P., et al., 2022 [27]
Sparsentan	ET_A_ * ~1000-fold	2005	Investigational	Promote proteinuria reduction Nephroprotective effects	Focal Segmental Glomerulosclerosis and IgA Nephropathy	Murugesan N., et al., 2005 [28] Komers, R., et al. 2020 [29]

* Dual ET_A_ and angiotensin-II type1 receptor antagonist. ^¥^ Year of development and status of each drug was searched in “NCATS Inxight Drugs” database in December 2022 [30].

**Table 3:** Renal effects displayed by selective endothelin receptor antagonists (ERAs) in different randomized controlled trials.

N	Study Population	Diabetes (%)	Baseline GFR (mL/min/1.73 m^2^)	Intervention/Control/ Follow-Up	Kidney Endpoints	Albuminuria/Proteinuria Reduction	BP Reduction (ERA-Placebo)	GFR Difference (ERA-Placebo)	Author/Study/Year
379	GFR > 30 mL/min/1.73 m^2^. Resistant hypertension.	153 (40%)	79.0	Darusentan 50, 100 or 300 mg/daily Placebo 3.5 months	NR	30.4 mg/g (UACR)	−9.9 mmHg in SBP(95%CI: −12.3–−5.7) −4.6 mmHg in DBP (95%CI: −7.0–−2.2)	−3.7 (95%CI: −6.9–−0.5)	Weber, M.A. et al. [91] 2009
1392	21–80 years. Creatinine 1.2–3 mg/dL. UACR ≥ 309 mg/g. Diabetic.	1392 (100%)	33.3	Avosentan 25 or 50 mg/daily Placebo 4 months	HR 0.87 (95%CI 0.6–1.2) ^a^	565.5 mg/g (UACR) 31.7% UACR reduction *	−5.1 mmHg in SBP−3.7 mmHg in DBP	0.15 (95%CI: −1.3–1.9)	Mann, J.F.E. et al. [36] ASCEND 2010
89	GFR > 20 mL/min/1.73 m^2^. UACR 100–3000 mg/g. Type 2 diabetes.	89 (100%)	52.8	Atrasentan 0.25, 0.75, 1.25 mg/daily Placebo 2 months	NR	27.5% UACR reduction * Not significant reduction with 0.25 mg	−8.2 mmHg in SBP−6.6 mmHg in DBPNot significant reduction with 0.25 mg	NR	Kohan, D.E. et al. [92] 2011
27	18–70 years. CKD stages 1 to 4. Non-diabetic.	0 (0%)	54.0	Sitaxsentan 100 mg/daily Placebo and nifedipine 30 mg/daily 1.5 months	NR	0.56 g/day (24-h proteinuria) 336.3 mg/g (UPCR)	≈−5 mmHg reduction in SBP and DBP	NR	Dhaun, N. et al. [93] 2011
211	>18 years. GFR 30 to 75 mL/min/1.73 m^2^. UACR 300–5000 mg/g. Type 2 diabetes.	211 (100%)	49.3	Atrasentan 0.75 or 1.25 mg/daily Placebo 3 months	NR	301.5 mg/g (UACR)	0.5 mmHg in SBP (95%CI: −5.0–6.0) 1 mmHg in DBP (95%CI: −2.8–4.8)	−0.5 (95%CI: −5.3–4.3)	Zeeuw, D. et al. [18] RADAR 2014
2648	18–85 years. GFR 25 to 75 mL/min/1.73 m^2^.UACR 300–5000 mg/g. Type 2 diabetes Responders (30% decrease in UACR).	2648 (100%)	43.9	Atrasentan 0.75 mg/daily Placebo 26.4 months	HR 0.65 (95%CI 0.5–0.9) ^b^	33.6% UACR reduction * (95%CI: 29.1–38.2)	−1.6 mmHg SBP reduction (95%CI: 0.7–2.5)	0.65 (95%CI: 0.3–1.0)	Heerspink, et al. [19] SONAR 2019
13	>18 years. Systemic sclerosis. CKD stages 2 to 3a.	0 (0%)	52.4	Zibotentan 10 mg/daily Placebo 6.5 months	NR	NR	NR	4.3 (95%CI: 2.6–11.3)	Stern, et al. [24] ZEBRA 1 2022

^a^ Doubling of serum creatinine, end-stage kidney disease or death. ^b^ Doubling of serum creatinine, end-stage kidney disease or death due to kidney failure. * UACR percentage reduction compared to place (the reduction observed in the placebo group has been subtracted to the reduction in the active treatment groups). BP: Blood Pressure; SBP: systolic blood pressure; DBP: Diastolic blood Pressure; CKD: Chronic Kidney Disease; GFR: Glomerular Filtration Rate; UACR: urine albumin-to-creatinine ratio; UPCR: urine protein-to-creatinine ratio; NR: not reported.

## Data Availability

Not applicable.
